# The impacts of the global gag rule on global health: a scoping review

**DOI:** 10.1186/s41256-019-0113-3

**Published:** 2019-08-29

**Authors:** Constancia Mavodza, Rebecca Goldman, Bergen Cooper

**Affiliations:** 1 Biomedical and Research Training Institute (BRTI), 10 Seagrave Rd, Avondale, Harare, Zimbabwe; 2 London School of Hygiene and Tropical Medicine (LSHTM), London, UK; 3 A Wider Circle, Silver Spring, USA; 4 Center for Health and Gender Equity, Washington, DC., USA

**Keywords:** Global gag rule, Mexico City policy, Global Health, Health systems, Abortion

## Abstract

**Background:**

The 1984 Mexico City Policy is a U.S. federal policy that has prohibited foreign nongovernmental organizations that receive U.S. international family planning assistance from using their own, non-U.S. funds to provide, counsel on, or refer for abortion services as a method of family planning, or advocate for the liberalization of abortion laws- except in cases of rape, incest, and life endangerment. The policy became known as the global gag rule (GGR) due to its silencing effect on abortion advocacy. Historically, it has only been attached to family planning funding, until 2017 when a presidential memorandum expanded the policy to nearly all US$8.8 billion in global health foreign assistance. In light of the aforementioned expansion, this scoping review aimed to describe and map the impacts of the GGR on global health, which in turn would identify research and policy gaps. This is the first time that all of the existing literature on the policy’s impact has been synthesized into one article and comprehensively reviewed.

**Methods:**

The review utilized Arksey and Malley’s five-stage methodological framework to conduct a scoping review. Fourteen peer-reviewed databases and 25 grey literature sources were searched for publications between January 1984 and October 2017. Organizations and individuals working on GGR research and impact were also contacted to access their works from the same time period. These publications reported on impacts of the global gag rule on 14 domains in global health.

**Results:**

The searches yielded 1355 articles, of which 43 were included. Overall, 80% of the identified sources were qualitative. The misunderstanding, miscommunication, and chilling effect of the policy underpinned the GGR’s impacts. The frequently reported impacts on family planning delivery systems (34 articles) and the loss of U.S. funding (21 articles) were often related. Sources reported on the impact of the GGR on HIV and AIDS programs, advocacy and coalition spaces, and maternal and child health. Only three studies (6.9%) quantified associations between the GGR and abortion rates, concluding that the policy does not decrease rates of abortion.

**Discussion:**

The GGR’s development and implementation was consistently associated with poor impacts on health systems’ function and outcomes. More peer-reviewed and quantitative research measuring and monitoring the policy’s impact on health outcomes are needed. More research and policy analysis exploring the GGR’s development and its implementation on the ground will improve knowledge on GGR consequences, and potentially shape its reform.

## Background

The Mexico City Policy (MCP) has significant impacts on global health and undermines already fragile health systems by disrupting system functions. System disruptions include loss of staff and resources and the reduction of health service provision for populations that need them. The MCP was instated in 1984 by President Ronald Reagan [1]. As a condition of receiving U.S. foreign assistance for family planning, the policy prohibits foreign non-governmental organizations (NGOs) from advocating for the liberalization of abortion laws; or counseling on, referring for, or providing abortion services as a method of family planning [1, 2]. Under the policy, abortion is permissible in the cases of rape, incest, life endangerment of the woman, and as a “passive referral”^1^ [2]. Since 1984, the policy has been enacted by every Republican president and rescinded by every Democratic president. The policy gags health providers from informing clients of their full range of reproductive options, as well as civil society organizations from advocating for legislative reform. Due to its gagging effect, the policy is often referred to as the Global Gag Rule (GGR), the term used throughout this article.

On January 23, 2017, President Donald Trump reinstated the GGR, renaming it “Protecting Life in Global Health Assistance” (PLGHA), and laying the groundwork for the expansion of the policy to nearly all forms of global health assistance. This includes funding for areas such as HIV and AIDS, maternal and child health (MCH), tuberculosis and malaria, gender-based violence (GBV), health systems strengthening, and water, sanitation and hygiene (WASH) [3].

There is a diverse body of work on past, current, and projected GGR impact, including research articles, projects, reports, and case studies, produced by a wide range of sectors including academic institutions, governments, and health and civil society organizations. A handful of peer-reviewed studies [4, 5] and grey literature pieces [6–8] have investigated the impact of previous implementations of the GGR on family planning programs. The expanded GGR has triggered documentation of how this policy has [9, 10] and will affect global health and health systems [11, 12].

As part of a larger policy and research report on the GGR, researchers from the Center for Health and Gender Equity (CHANGE)^2^ designed a scoping review that assembles existing evidence on the impact of the GGR on health systems from 1984 to 2017 [13]. This is the first time that all of the existing literature on the policy’s impact has been synthesized into one article and comprehensively reviewed. There is sufficient evidence to determine that the GGR is harmful and that there is insufficient existing documentation of all the harms of the policy. Consequently, there is a fragmented understanding of the scope of the GGR’s impacts. This constrains knowledge generation for policy development and implementation and underestimates the ripple effect that the policy has had across health system areas.

Facilitating a full mapping and understanding of what is known about the GGR’s impacts is critical because it can:

- Identify gaps in evidence generation;
- Reveal how the GGR is conceptualized and understood by the diverse stakeholders interacting with the policy;
- Inform construction of policy for effective health service delivery.

This article outlines the scoping review methodology and the consequent mapping of evidence on the policy’s impacts to address the objectives stated above. A discussion on the key findings in relation to evidence generation, existing understanding of the policy, and policymaking is also offered.

## Methods

This review followed Arksey and Malley’s five-stage methodological framework: (1) identifying the research question; (2) identifying relevant studies; (3) study selection; (4) charting the data; and (5) collating, summarizing, and reporting the results [14]. A scoping review methodology was adopted as it aims to identify, map, and synthesize key concepts on broad topics, without assessing the quality of the included literature- as would be the case for a systematic review [15]. Currently, there is a dearth of empirical evidence and research on the GGR; and most of the evidence is from non-academic sources as will be seen in the findings of this review. Therefore, the scoping review methodology is most appropriate for mapping the evidence of the GGR’s impact. In this research, “impact” is defined as a change or consequence and “health systems” include health care: institutions, resources, services and programs, civil society, advocacy work, providers, health outcomes, and the individuals, and communities served [16].

### Identifying the research question

The preliminary research question for this review was: *What is the impact of the Global Gag Rule on health systems?* The broad nature of this question was intended to capture the potential breadth of the GGR’s impact since its inception, and as well as any impacts recorded since the policy’s expansion. CHANGE researchers identified 17 health system focus areas for the review.

### Literature search strategy

A three-step literature search process was performed to exhaustively capture the existing evidence of GGR impact. The established GGR key terms were “Global Gag Rule,” “Mexico City Policy,” and “Protecting Life in Global Health Assistance.” Key and MeSH terms were also established for the selected domains. In the peer-reviewed literature search (Table 1), the GGR key terms and the selected domains’ (Table 2) key terms were combined using the Boolean term “AND” in all the electronic databases explored (see Table 7 in Appendix).

**Table 1:** Peer-Reviewed Literature Electronic Database Sources

• BioMed Central (BMC)	
• Google Scholar	
• The Lancet	
• Population Information Online (POPLINE)	
• PsychINFO	
• Public Library of Science (PLOS)	
• PubMed (ie. MEDLINE)	
• ScienceDirect	
• Scopus	
• Sociological Abstracts (Proquest)	
• UNICEF ChildInfo database	
• Web of Science	
• Wiley Online Library	
World Health Organization Institution Repository for Information Sharing (WHO IRIS)

**Table 2:** Global Health Domains Searched

1. Abortion	
2. Advocacy	
3. Commodities (male and female condoms, emergency contraception, pre- and post-exposure prophylaxis)	
4. Family planning	
5. Gender-based violence (GBV)	
6. Global health assistance	
7. HIV and AIDS and STIs	
8. Human rights	
9. Infectious diseases	
10. Key populations	
11. Maternal morbidity and mortality	
12. Maternal and child health (MCH)	
13. Non-communicable diseases (NCDs)	
14. Orphans and vulnerable children (OVC)	
15. Prevention of mother-to-child transmission (PMTCT)	
16. Reproductive health	
17. Water, sanitation and hygiene (WASH)	

For the grey literature search, each key term was put into the 25 established websites’ publication databases (Table 3) when available, and general search bars when necessary. Different websites required a different number of tab selection, and a unique search strategy was used for one source due to its website format, which required the selection of “Global Gag Rule” from a dropdown menu within its publications tab. In five of the websites, no publications were obtained after using the key terms and search strategy.

**Table 3:** Grey Literature Sources

• amfAR, The Foundation for AIDS Research	
• Asian-Pacific Resource and Research Centre for Women (ARROW)	
• Bixby Center for Global Reproductive Health, University of California San Francisco	
• Center for Reproductive Rights (CRR)	
• The George Washington University	
• The Global Women’s Institute (GWI), The George Washington University	
• Guttmacher Institute	
• Human Rights Watch (HRW)	
• Ibis Reproductive Health	
• Institute for Reproductive Health (IRH), Georgetown University	
• International Center for Research on Women (ICRW)	
• International Women’s Health Coalition (IWHC)	
• Ipas	
• Kaiser Family Foundation (KFF)	
• London School of Economics (LSE)	
• The London School of Hygiene and Tropical Medicine (LSHTM)	
• Mailman School of Public Health, Columbia University	
• Management Sciences for Health (MSH)	
• O’Neill Institute for National and Global Health Law, Georgetown University	
• PAI	
• Population Council	
• Rutgers International	
• UAB School of Public Health	
• The Williams Institute, UCLA School of Law	
• Yale School of Public Health	

Finally, listservs, coalition groups of organizations, and individual researchers known to be doing work on the GGR were contacted to request their work for review inclusion. Additionally, after identifying one institution doing its own scoping review of GGR literature, search results were compared to identify research gaps.

### Inclusion and exclusion criteria

To address time constraints and focus searches, literature was only included if it was available in English and published between 1984 and 2017. Inclusion and exclusion criteria for this review were established and implemented. Inclusion criteria were peer-reviewed journal research articles, organizational reports, working papers, master’s theses, and accessible book chapters. Exclusion criteria were fact sheets, policy briefs, blog posts, news articles, press releases, newsletters, opinion pieces, toolkits and advocacy guides, infographics, videos, letters, and transcriptions. Policy briefs were included if they had original findings, such as PAI’s case studies of GGR impact within countries, which were internally classified as policy briefs.

### Study selection

**Fig. 1:**
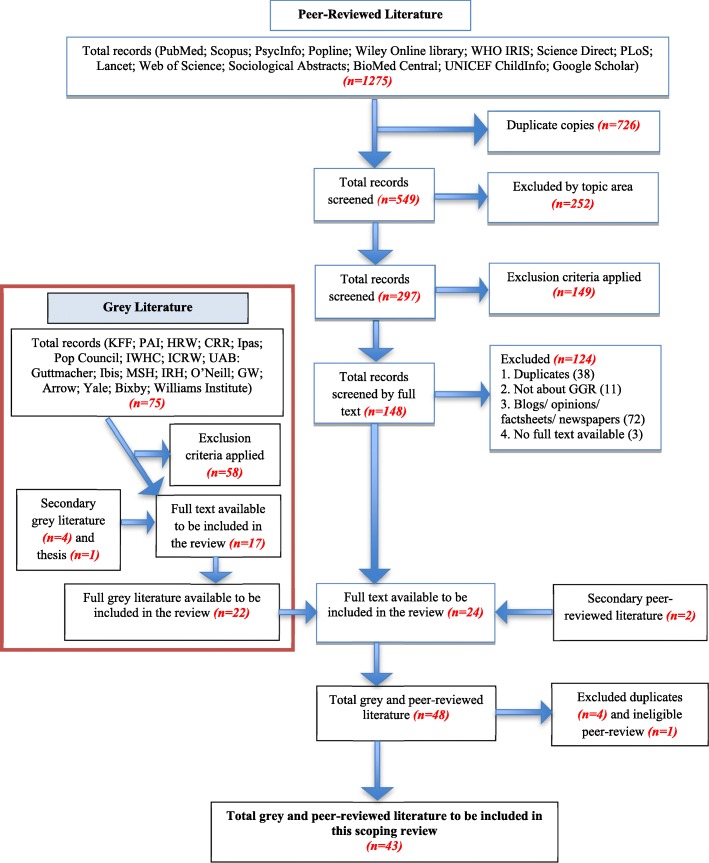
Search Flow Chart

The peer-reviewed search strategy identified 1275 articles. Duplicate copies were removed and the remaining articles were screened for relevance by topic area. The established inclusion/exclusion criteria were applied to 297 articles, 148 of which were selected for further screening. Of these articles, the three that did not have full text accessible were removed, leaving 145 articles. After a full-text reading, an additional 121 articles did not meet the inclusion criteria, and the remaining 24 articles were included in this review. Two additional articles from colleagues were identified and included, resulting in a total of 26 articles for review inclusion (Fig. 1).

The grey literature search strategy identified 75 articles. These were screened using the established inclusion/exclusion criteria and for relevance to yield 17 articles. Four additional eligible articles were identified by colleagues also doing GGR research, and one master’s thesis was discovered after the review data collection period, resulting in a total of 22 articles for review inclusion.

All the articles that addressed GGR impact were included, regardless of methodological approach. An article was excluded if it referenced or talked about the GGR without addressing its impact or implications. For example, a Human Rights Watch report on the lack of access to abortion in Peru defined the GGR and recommended that the United States Agency for International Development (USAID) clarify the policy for the Peruvian government but did not link the GGR to abortion access or other health system indicators, so this article was excluded from the review. The 26 peer-reviewed and 22 grey literature articles were combined, duplicates were removed, and after consultation with an author, one peer-reviewed article was removed due to corrupt data. The peer-reviewed search pulled some pieces that were reports and classified as grey literature. Resultantly, 43 articles addressing the impact of the GGR were included in this scoping review.

### Charting the data

An excel spreadsheet was used as the data extraction summary form to collect general citation information, study type and methodology, country and population of focus, study approach, and key findings on policy impact.

### Data collation, analysis, and synthesis

All 43 articles were read at least twice. CM manually coded and discussed emerging themes with RG and BC. To manage the breadth of the research question and the volume of literature uncovered, narrative descriptive synthesis was used and the findings were classified using the established focus areas (Table 2), allowing for the inductive identification of themes [17]. The focus areas and emerging themes gave structure to the key findings.

## Results

The 43 articles in this review include 16 peer-reviewed publications and 27 grey literature materials (Table 4). Thirty-four pieces are qualitative, and the 9 quantitative include: 3 peer-reviewed publications, one of which looks at the relationship between the GGR and sub-Saharan Africa abortion rates [5], one at donor money allocation, [47] and the third at the relationship between contraceptive supplies and fertility outcomes during GGR years [34]; one working paper on family planning aid in developing countries [18]; a country-specific study on the impact of the GGR on unintended pregnancy, abortion rate, and child health [4]; and a book chapter on the impact of the GGR on abortion rates in four global regions [48]. The remaining 3 quantitative studies are master’s theses [24, 30]. Eighteen articles come from just three organizations working in global health. The dominant qualitative approach is a case study, and the quantitative works are largely regression analyses [4, 5]. Less than half of the literature focuses on specific countries. Most of the literature (86%) discusses the previous enactments of the GGR and only 7 of the 43 articles are on PLGHA. The reported impacts of the GGR are on: global health assistance, reproductive health services and outcomes, family planning programs, contraceptive supplies and demand, abortion rates, HIV and AIDS programs and rates, civil society participation, NGO political advocacy, and human rights.

**Table 4:** Summary Table of Articles Included in Review

Authors/(Year) (Title)	peer review/grey lit	Country/region focus	Type of Study and methods	Approach	Key findings on the Impact of the GGR
Asiedu, E., Nanivazo, M., & Nkusu, M, (2013) [18]: Determinants of foreign aid in family planning: How relevant is the Mexico City Policy?	Working paper unpublished (grey lit)	151 developing countries	Quantitative (difference and system GMM estimators): panel data from WDI and OECD	Analyze the determinants of Family Panning (FP) Aid and examine the extent to which US foreign policy on FP affects the allocation of FP aid to developing countries- particularly examining the effect of MCP on the allocation of FP aid to developing countries	Impact on: Family Planning (FP AID, all things equal, decreased by 3–6% during Bush-era policy years)
Bendavid, E., Avila, P., & Miller, G. (2011) [5]: United States aid policy and induced abortion in sub-Saharan Africa	Peer- review	20 African countries	Quantitative difference-in-difference analysis: DHS data from 261,116 women aged 15–44 yrs.	Determine the relationship between the reinstatement of MCP and the probability of a woman in SSA having an induced abortion rate	Impact on: abortion rates (GGR was associated with a 2.55 greater increase in the odds of self-reported abortion in high MCP-exposure countries vs. low MCP-exposure countries); Modern contraceptives (relative decline in contraceptive use in high exposure countries)
Bingenheimer, J. B., & Skuster, P. (2017) [11]: The Foreseeable Harms of Trump’s Global Gag Rule	Peer- review commentary	n/a	Qualitative analysis (commentary): summarizes and analyzes impacts of the GGR up until 2017	Provide a scientific and policy basis for the three criticisms of the GGR: (1) that the rule fails to accomplish its presumed objective of reducing the number of abortions; (2) that it negatively affects the health and well-being of individuals and populations in affected countries; and (3) that it interferes with governments’ ability to meet their international obligations.	Impact on: maternal deaths (premised linkage associations); FP programs disrupted; child health poor; socio-economic developments are poor; contraceptive use; abortion rates; fertility; loss of funding; abortion rates; advocacy
Blane, J., & Friedman, M. (1990) [19]: Mexico City Policy implementation study	Organizational report	5 Cooperating Agencies (FHI; Pathfinder; IPPF/WHR; Center for Development and Population Activities; and the Association for Voluntary Surgical Contraception) and 49 subgrantees of theirs in 6 countries- (10 in Pakistan, 8 Bangladesh, 12 Brazil, 8 Kenya, 4 Egypt, 7 Turkey)	Qualitative interviews; site inspections; document reviews & analysis	Determine whether recipients of grants and their sub-grantees are in compliance with MCP 2) determine whether the standard clause is understood by the grantees & subgrantees 3) determine what impact if any the MCP has had on FP programs	Impact on: advocacy; partnerships & coalitions; provider-client interactions; confusion & poor policy understanding; poor communication; chilling effect (self-censoring & over-caution);
Bogecho, D., & Upreti, M. (2006) [20]. (CRR): The Global Gag Rule--an antithesis to the rights-based approach to health	Commentary peer review	Kenya & Nepal	Qualitative narrative: based on country case findings	Summarizing the impact of the GGR in Nepal & Kenya. In Kenya, the GGR impact was on health services. In Nepal, the GGR impact was on human rights.	Impact on Kenya: loss of funding for FPAK & MSI; severe disruption of FP services (closed 8 clinics (6 FPAK & 2 MSI)- 28,000 clients were denied health services, fire over 30% of their staff & cut back services); HIV prevention efforts like VCT & HIV prevention services were severely restricted. Impact on Nepal: Rights to health, life & information is hampered: loss of funding (FPAN suffered budget setbacks for rejecting the GGR); FP services disrupted (clinics were closed & staff laid off overnight); government impact (government liberalized the abortion law and was reliant on NGOs to provide abortion services, and track implementation of the new law, they were not able to do so once FPAN did not sign the GGR.
Camp, S. (1987) [21]: The impact of the Mexico City Policy on women and health care in developing countries	Peer-review commentary	8 developing countries & the U.S.A	Qualitative narrative analysis: Reporting on interviews with family planning organizations in the US and 8 developing countries	Review the development & scope of the MCP as well as present preliminary findings of ongoing research evaluating the impact of MCP on foreign FP and population assistance programs	Impact on: loss of funding; confusion & poor policy understanding; increased administrative burden; chilling effect (self-censoring & overcautiousness); partnerships & coalitions;
Carroll, L (2012) [22] / University of North Carolina at Chapel Hill: The Effects of the Mexico City Policy on Antenatal Care and Skilled Birth Attendance in Developing Countries	Master’s Thesis	37 developing countries	Quantitative analysis: DHS data from 98 surveys between 1998 and 2008 for 251,602 women ages 15–49 years	To determine if women in countries with a high degree of exposure to the Mexico City Policy have diminished odds of having effective antenatal care and skilled birth attendance at delivery compared to women in countries with a lower degree of exposure to the policy	Impact on maternal health (skilled birth attendance & antenatal care): outcomes improved in most parts of the world between 1993 and 2000 and 2001–2008 regardless of exposure to the policy.
Chávez, S., & Coe, A.-B. (2007) [23]: Emergency Contraception in Peru: Shifting Government and Donor Policies and Influences	Peer-review	Peru	Qualitative narrative analysis: Reporting on information gathered from on-going monitoring conducted by authors of reproductive health and rights policies in Peru	Analyze the trajectory and reasons for several shifts in USAID/Peru’s policy on EC within the context of changes in government policy on FP in both countries	Impact on: Advocacy for liberal abortion laws (due to poor understanding of the policy anti-choice groups leveraged the GGR to stop the USAID/Peru from engaging in Emergency contraceptive provision)
Choudhury, S./ Georgetown University (2012) [24]: Quantitative analysis: DHS data for individual women in Ghana	Master’s Thesis	Ghana	Quantitative analysis: DHS data (1998 & 2001) for individual women and men in Malawi	Examine the effect of the Mexico City Policy on access to modern contraceptive methods, prenatal care, and delivery assistance	Impact on: Bush-era GGR had negative effects on prenatal care access in rural and urban areas.
Crane, B. B., & Dusenberry, J. (2004) [25]: Power and politics in international funding for reproductive health: the US Global Gag Rule	Peer- review	n/a	Qualitative narrative analysis: describes the GGR and its impacts- especially the Bush-era GGR.	Reviews the history and political origins of the Gag Rule under several Republican party presidents, including its roots in US domestic abortion politics, and analyses the short and long-term damage the Gag Rule is causing to the health and lives of women in the developing world.	Impact on: loss of funding (IPPF & MSI); human rights & autonomy; lack of transparency (USAID did not provide information on policy implementation); abortion (not reduced abortions); projected HIV and AIDS impacts; separating partnerships between ‘gagged’ and ‘ungagged’; contraceptive supply; chilling effects
Crimm, N. J. (2007) [26]: The Global Gag Rule: Undermining national interests by doing unto foreign women and NGOs what cannot be done at home	Peer- review	n/a	Qualitative narrative analysis: narrates an analysis of the harms to human rights, autonomy, reproductive health and family planning of GGR	Analysis that focuses on the GGR’s grave harm to U.S. national interests; human rights & autonomy of foreign women; family planning and reproductive health	Impact on: human rights & autonomy; women’s rights (oppressed women by controlling and restricting their access to information and health care); chilling effects; family planning and reproductive health (the GGR has barrelled and even hampered the work/efforts of foreign NGOs & their HWs in providing RH and FP services); clinic closures; freedoms of speech & association, are silenced.
CRR (2009) [27]: Expanded Global Gag Rule Limits Women’s Rights and Endangers Their Well-being	Report	14 Countries (Kenya, Nigeria, Mozambique, Ethiopia, Rwanda, Haiti, Tanzania, Uganda, Cote D’Ivoire; Zambia, Namibia, Botswana, Guyana, South Africa)	Qualitative descriptive summary & analysis of direct and potential GGR effects	Describes GGR expansion under the Bush administration, and uses analysis of abortion laws in 14 countries to reveal harmful GGR effects	Impact on: Loss of funding; abortion, advocacy, FP, GBV, global health assistance, HIV/AIDS & STIs, maternal mortality, reproductive health; free speech silenced
Curtis, C., Farrell, B., & Ahlborg, J. (2005) [28]: *Cambodia Postabortion Care:* Cambodia Postabortion Care Program. Final report of findings and recommendations. Cambodia trip report: dates April 25, 2005, to May 6, 2005	Report (grey)	Cambodia	Qualitative descriptive narrative analysis: interviews with staff from USAID/ Cambodia; CARE; RHAC; RACHA; the Ministry of Health; and Directors of operational districts. Site visits to health centers, community education sessions; and the Red Cross Hospital. Documents review of national policy documents; PAC curriculums; service delivery guidelines; USAID/ Cambodia post-abortion care draft strategy	USAID report: an assessment of Cambodia’s Post Abortion Care Program & compliance to the Bush-era Mexico City Policy conducted by USAID/Washington	Impact on: Post Abortion Care (PAC)- Cambodia was in compliance with the GGR: Potential maternal health impact; Confusion- CARE/Cambodia had entered a contract with MOH & was unsure at what point they will violate the GGR; Providers at RHAC were trained on GGR; lack of clarity- questions regarding the MCP and separation of abortion care vs. post-abortion care especially in rural health centers were the usually is only one midwife providing all types of care.
Ernst, J., & Mor, T. (CRR) (2003) [29]: Breaking the Silence: The Global Gag Rule’s Impact on Unsafe Abortion	Report	Ethiopia, Kenya, Peru, Uganda	Qualitative narrative & overview analysis: interviews with a broad range of reproductive health, U.N. and government actors in 4 countries (25–30 interviews per country)	Documenting Bush-era GGR impacts on organizations normally receiving USAID family planning funding	Impacts on: loss of funding, abortion, advocacy, commodities (EC), FP, global health assistance, HIV/AIDS & STIs, human rights, infectious diseases (immunizations), key populations (AGYW), maternal mortality/morbidity, reproductive health
Foster, S. C./ Georgetown University (2009) [30]: Trends in condom use: The association in Malawi of condom use with AIDS knowledge and the relationship to the global gag rule	Master’s Thesis	Malawi	Quantitative analysis: DHS data (2000 & 2004) for individual women and men in Malawi	Examines the relationship between the characteristics of individual Malawians and their condom use over time, and evaluates the impact of GGR overtime	Impact: There is a positive relationship between AIDS knowledge and condom use in Malawi. The GGR has a negative effect on condom use over time;
Fox, G. H. (1985) [31]: American population policy abroad: The Mexico City abortion funding restrictions	Peer review	n/a	Qualitative descriptive narration & analysis	Historical context on the legalities surrounding the GGR with a policy/legislative focus.	Impact on: loss of funding, lack of clarity in how to implement 1985 GGR, AID had not yet come up with the abortion funding restrictions for the GGR. All funding to UNFPA was terminated & it remained unclear which organizations would be impacted. MCP imposes a view on abortion which many NGOs do not share.
Gezinski, L.B. (2011) [32]: The Global Gag Rule: Impacts of a Conservative Ideology on Women’s Health.	Peer-review	n/a	Qualitative literature review & narrative policy analysis	Outline the legislative history of the Global Gag Rule and will describe the key stakeholders responsible for the policy’s passage and promotion	Impact on: human rights; political advocacy; law; reproductive health; abortion, perceived and real loss of funding; and the health system effects that were associated with that (decreased access to contraceptives, increased rates of unwanted pregnancies, as well as increased abortions resulting in harm or death
Jones, A. A. (2004) [33]: The “Mexico City Policy” and Its Effects on HIV / AIDS Services in Sub-Saharan Africa	Peer review	Sub-Saharan Africa (Uganda, Kenya, Ethiopia)	Qualitative thematic- narrative & analysis	Analyze and show the impact of GGR on HIV/AIDS programs in Sub-Saharan Africa.	Impact on: loss of funding HIV/AIDS Services that are provided by Family Planning clinics (Health centers were closed down −17 Uganda, 5 Kenya, 1 Ethiopia). HIV/AIDS services are usually integrated into FP programs; advocacy coalitions were disbanded; human rights violation; legal rights of women with HIV
Jones, Kelly, M (2015) [34]: Contraceptive Supply and Fertility Outcomes: Evidence from Ghana	Peer-review	Ghana	Quantitative analysis: DHS data for individual women in Ghana	To examine Ghanaian women’s response to a reduction in the availability of modern contraceptives in terms of contraceptive access and use, resulting pregnancies, use of induced abortion, and resulting births. The exogenous change in availability is due to GGR-related loss of funding and the associated outcomes of this loss.	Impact on: Loss of funding (PPAG agreed to sign the MCP to keep ¼ of its budget that was USAID funded for the CBS project; PPAG funding from IPPF (75% of its budget) was reduced by 54%); contraception (contraceptive ability was reduced during policy periods which affected contraceptive use); family planning programs (PPAG closed down 57% of its clinics).
Jones, K. M. (2011) [4]: Evaluating the Mexico City Policy: How US foreign policy affects fertility outcomes and child health in Ghana	Report (grey)	Ghana	Quantitative analysis (estimation employing woman-by month panel of pregnancies & woman-fixed effects): DHS MEASURE data for Ghanaian women	Estimate whether a given woman is less likely to abort a pregnancy during two policy periods versus two non-policy periods.	Impact on: abortion (Regressive effects: increased (200000) abortions in a rural area); additional 12% unintended pregnancies and 1/2 to 3/4 million additional unintended births due to loss of contraceptive health services; as well as dilapidated child health in height and weight for age for children born from these unintended births. The GGR does not achieve its aim for women in the Ghana context.
Law, S. A., & Rackner, L. F. (1987) [35]: Gender Equality and the Mexico City Policy	Peer review	n/a	Qualitative descriptive narration & analysis	Provides comparative historical contexts of the legalities and constitution surrounding the MCP as well as gender (equality). Then provides a gender equality analysis of the MCP	Impact on: women’s rights- discriminates against women & their health providers by not providing all information to make fully informed choices; family planning; abortion; freedom of choice- the GGR disrespects choice and dignity of women they cannot choose to bear or not bear children: the GGR fails on equality
Leitner Center for International Law and Justice / Fordham Law School (2010) [36]: Exporting Confusion: U.S. foreign policy as an obstacle to the implementation of Ethiopia’s liberalized abortion law. New York: Fordham Law School	Report	Ethiopia	Qualitative interviews & analysis: women, donors, providers, NGO & government staff,	Examines the effects of US foreign assistance and policies on Ethiopia’s attempts to deal with unsafe abortions, high maternal mortality and liberal abortion laws	Impact on: Ethiopia’s attempt to address high rates of unsafe abortion through the liberalization of its abortion law; GGR has negatively affected the availability of comprehensive safe abortion services for Ethiopian women; US exported domestic debate about abortion to Ethiopia
Neier, A. (1987) [37]: The right to free expression under international law: implications of the Mexico City Policy	Peer-review	n/a	Qualitative thematic descriptions & analysis	Explore how the MCP is a violation of international freedoms of expression, speech, and opinion in the context of the US ratifying and adopting these international declarations	Impact on: the freedom of expression and opinion- human rights (implementing the GGR means the US is violating internationally recognized freedom of expression rights); chilling effects
Nowels, L. (2001) [38]: International family planning: the “Mexico City” policy. Updated April 2, 2001. CRS Report for Congress.	Report (grey)	n/a	Qualitative thematic descriptions & analysis	Reviews the experience of the original “Mexico City” policy between 1984 and early 1993: provides background on the 1984 decision (policy implementation, legal challenges, funding reallocation, an assessment of the impact and implications of the Mexico City policy, and a summary account of congressional efforts to modify the policy	Impact on: Loss of funding (IPPF); court challenges delayed GGR clauses being attached for some organizations; Family planning AID (The US said it would keep the Family Planning aid amounts the same and reallocate funds that would otherwise have gone to IPPF/London ($16.5mil) and UNFPA (10mil) in the 1985 version implementation, to organizations that certify for the GGR).; Lack of clarity & confusion.
PAI (2005) [38, 39]: Access Denied – The Impact of the Global Gag Rule in Ethiopia	Case Study Report	Ethiopia	Qualitative interviews with key informants (women, girls, health providers, NGO & government staff) & analysis	Describes and analyses the direct and potential threats of the G.W. Bush-era GGR in Ethiopia	Impact on: Loss of funding, the GGR has resulted in the loss of technical assistance and contraceptive donations to key NGOs in Ethiopia, worsening the country’s supply shortage. CBD programs were shut down, condom corners were closed. Misinformation and incorrect policing of HIV/AIDS programs by GGR were widespread. Some organizations could no longer advocate for more liberal abortion laws in Ethiopia
PAI (2005) [38, 39]: Access Denied – The Impact of the Global Gag Rule in Ghana	Case Study Report	Ghana	Qualitative interviews with key informants (women, girls, health providers, NGO & government staff) & analysis	Describes and analyses the direct and potential threats of the G.W. Bush-era GGR in Ghana	Impact on: loss of funding; disrupted key reproductive health programs; cut back essential rural outreach activities and clinic services; dismantled partnerships between reproductive health organizations and HIV/AIDS activities.
PAI (2005) [38, 39]: Access Denied – The Impact of the Global Gag Rule in Kenya	Case Study Report	Kenya	Qualitative interviews with key informants (women, girls, health providers, NGO & government staff) & analysis	Describes and analyses the direct and potential threats of the G.W. Bush-era GGR in Kenya	Impact on: loss of funding-Reproductive Health (RH) care deteriorated, fertility increased, contraceptive uptake stagnated, medically assisted birth rate plummeted. The GGR exacerbates an already worse reproductive health situation; clinics closed down; staff laid off; splintered integrated services
PAI (2005) [38, 39]: Access Denied – The Impact of the Global Gag Rule in Nepal	Case Study Report	Nepal	Qualitative interviews with key informants (women, girls, health providers, NGO & government staff) & analysis	Describes and analyses the direct and potential threats of the G.W. Bush-era GGR in Nepal	Impact on: loss of funding; Innovative RH programs have been terminated; Free speech, expression and opinion for the democratic liberalization of abortion laws curtailed; Government’s sovereignty infringed upon
PAI (2005) [38, 39]: Access Denied – The Impact of the Global Gag Rule in Tanzania	Case Study Report	Tanzania	Qualitative interviews with key informants (women, girls, health providers, NGO & government staff) & analysis	Describes and analyses the direct and potential threats of the G.W. Bush-era GGR in Tanzania	Impact on: Loss of funding - two major FP organizations in Tanzania withdrew critical technical support for the government’s FP programs; worsening contraceptive supply problems; rural clinics were closed; Key staff were lost/fired
PAI (2005) [38, 39]: Access Denied – The Impact of the Global Gag Rule in Zambia	Case Study Report	Zambia	Qualitative interviews with key informants (women, girls, health providers, NGO & government staff) & analysis	Describes and analyses the direct and potential threats of the G.W. Bush-era GGR in Zambia	Impact on: already struggling FP and RH provision is exacerbated; staff laid off and reduced services; HIV prevention efforts deprioritized which inhibits of HIV/AIDS + FP integration efforts.
PAI (2005) [38, 39]: Access Denied – The Impact of the Global Gag Rule in Zimbabwe	Case Study Report	Zimbabwe	Qualitative interviews with key informants (women, girls, health providers, NGO & government staff) & analysis	Describes and analyses the direct and potential threats of the G.W. Bush-era GGR in Zimbabwe	Impact on: loss of funding; restricted critical partnerships addressing both FP and HIV/AIDs; reduced family planning funds; (Brooke- Alexander Amendment + GGR); scaled back family planning programs with staff layoffs
PAI (2017) [9, 10, 40]: The Global Gag Rule & Maternal Deaths Due to Unsafe Abortion	Report	Various countries as examples	Qualitative narrative analysis	Describes and analyses the direct and potential threats of the Trump-era expanded GGR on unsafe abortions and maternal mortality	Impact on: advocacy to reduce injuries and maternal deaths caused by unsafe abortions in countries receiving U.S. aid; silences free speech and national sovereignty, and discourages democratic debate on abortion law reform in Kenya, Ethiopia, Mozambique, Uganda, Nigeria; hinders the efforts of governments and NGOs in seeking international assistance to implement new, liberalized abortion laws. The GGR contradicts international agreements.
PAI (2017) [9, 10, 40]: The Global Gag Rule & Contraceptive Supplies	Report commentary	n/a	Qualitative narrative analysis	Describes and analyses the direct and potential threats of the Trump-era expanded GGR on contraceptive supplies	Impacts on: Organizations that don’t sign the GGR also lose access to U.S.-donated contraceptives, including condoms, which enable women and men to prevent unintended pregnancy, protect themselves from HIV/AIDS, and avoid unsafe abortion (a leading cause of maternal injury, illness, and death in the developing world).
PAI (2017) [9, 10, 40]: Trump’s Global Gag Rule and Ethiopia	Report	Ethiopia	Qualitative descriptions summary (from fact-finding) & analysis	Describes and analyses the direct and potential threats of the Trump-era expanded GGR	Impact on: loss of funding; contraceptive supply distribution for two largest in-country contraceptive service delivery organizations (Family Guidance Association of Ethiopia and Marie Stopes International Ethiopia)- could increase Ethiopia’s unmet need, which currently stands at 22.3%; Progress on improving maternal mortality is likely to stall; miscommunication around the GGR could impact the implementation of liberalized abortion law in Ethiopia. PLGHA may scale back condom distribution; deteriorate integrated services, increasing the difficulty of accessing comprehensive health services. The impact of PLGHA will be disproportionately felt by young, rural, and poor women and girls.
PAI (2017) [9, 10, 40]: Trump’s Global Gag Rule and Senegal	Report	Senegal	Qualitative descriptions summary (from fact-finding) & analysis	Describes and analyses the direct and potential threats of the Trump-era expanded GGR in Senegal	Impact on: risk of losing funding; threatening increase the already high 25% unmet contraceptive need in the country; GGR places close to $8.5mil of SRHR funds at risk in Senegal (either programs will be delayed, toned down or closed). Abortion laws in Senegal are restrictive & GGR may increase the 63% unsafe abortions; increase confusion + fear surrounding implementation restrictions & conditions
Seevers, R. E. (2005) [41]: The Politics of Gagging: The Effects of the Global Gag Rule on Democratic Participation and Political Advocacy in Peru.	Peer review	Peru	Qualitative narratives & analysis	Examine the damaging effects of the Global Gag Rule on civil participation and political advocacy by NGOs focusing on reproductive rights in Peru and the overall effect this may have on the country’s emerging conception of democracy	Impact on: disrupted democracy and advocacy processes in Peru. Peru’s priorities included active civil society and NGOs engagement. The same NGOs that were being engaged for democracy & governance were also being gagged by the GGR- free speech, opinion and expression were heavily regulated
Skuster, P. (2004) [42]: Advocacy in whispers: the impact of the USAID Global Gag Rule upon free speech and free association in the context of abortion law reform in three East African countries	Peer review	Uganda, Ethiopia, Kenya	Qualitative interviews with key informants (NGOs, Government Officials) in Kenya, Uganda & Ethiopia & Thematic analysis	To study the effect of the GGR upon the free speech and free association of advocates of access to safe abortion	Impact on: coalitions and partnerships that have political authority and power; advocacy- the GGR curtails the ability of the reproductive health community to bring information on the effect of the restrictive law to lawmakers; chilling effect & silencing; promoted anti-choice narratives; splinters coalitions/ partnerships
Sneha Barot & Susan A. Cohen / Guttmacher (2015) [43]: The Global Gag Rule and Fights over Funding UNFPA: The Issues That Won’t Go Away	Report	Lesotho and Madagascar	Qualitative analysis synthesizing GGR effects	Descriptive synthesis of GGR-related loss of funding and its impacts on family planning as well as analysis of UNFPA funding cuts.	Impact on: legal challenges (Clinton administration); abortion, contraceptive supplies; family planning programs, loss of funding, chilling effect of the GGR continues even in non- policy years
Sneha Barot/ Guttmacher (2017) [44]: When Antiabortion Ideology Turns into Foreign Policy: How the Global Gag Rule Erodes Health, Ethics, and Democracy	Report	n/a	Qualitative analysis synthesizing GGR effects on health, ethics, and democracy	descriptive synthesis of GGR-related impact on loss of funding, health, ethics, and democracy	Impact on: Abortion, advocacy for liberal abortion laws, global health assistance, HIV/AIDS & STIs, human rights, diseases (immunization): confusion & lack of clarity; disrupted health programs; disintegrated health programs; fractured partnerships between US NGO and their local partners, and coalition spaces; hindered free speech, expression, and opinion
Susan A. Cohen/ Guttmacher (2009) [45]: The Reproductive Health Needs of Refugees and Displaced People: An Opening for Renewed U.S. Leadership	Report	n/a	Qualitative description and analysis	Describes the effect of US foreign policy including GGR on the reproductive needs of refugees and displaced populations	Impacts on: coalition spaces (terminated the RHRC consortium)
Susan A. Cohen/ Guttmacher (2011) [46]: U.S. Overseas Family Planning Program, Perennial Victim of Abortion Politics, Is Once Again Under Siege	Report	n/a	Qualitative description and analysis	Examines established impacts of the GGR recap/citations & analysis	Impacts of the GGR: it does not reduce abortions; effective family planning programs closed down, contraceptive supply distribution is disrupted; silencing of advocacy around unsafe abortions
van Dalen, H. P. (2008) [47]: Designing Global Collective Action in Population and HIV/AIDS Programs, 1983–2002: Has Anything Changed?	Peer review	n/a	Quantitative analysis: Panel data on expenditures of OECD donors for three types of aid agencies (multilateral, NGOs, and bilateral aid) for the years 1983–2002	Explore which forces might be relevant in explaining the provision of foreign aid through the set of aid channels toward family planning and HIV/AIDS programs	Impact on other donor funding: the GGR implementation years had no visible aggregate effect on other donors’ levels of funding
Yana van der Meulen Rodgers/ Rutgers University (2018) [48]: Impact of the Gag: New Estimates	Book Chapter	Latin America and the Caribbean, Eastern Europe and the Middle East, South and Southeast Asia, and Sub-Saharan Africa	(unpublished work) Quantitative analysis-regression analysis using DHS data	Determine the relationship between the reinstatement of G.W. Bush GGR induced abortion rate for women in 4 global regions	Impact on induced abortion rates: Women in Latin America and the Caribbean, highly exposed to the GGR, had more than 3 times the odds of having an abortion, compared to women in less exposed countries & before policy reinstatement. In SSA, Women highly exposed to the GGR had about 2 times the odds of having an abortion, compared to women in less exposed countries & before policy reinstatement. In Eastern Europe and the Middle East, and in South and Southeast Asia, the odds of having an abortion declined in highly exposed countries after reinstatement, compared to low exposed countries. The relationship between strict abortion laws & women’s likelihood of having an abortion needs further research.

### Misunderstanding the GGR

Foreign NGOs to whom the GGR applied were confused about the policy [19, 23, 49]. During the Reagan policy years, prime partners in Kenya and Bangladesh were unclear about the practical implementation of the policy, including the permissibility of post-abortion care and the repercussions of non-adherence [19, 38]. During a study visit to Kenya at the time, over 64% of implementing clinicians interviewed reported that the policy had never been explained to them [19].

Compared to prime non-implementing organizations, sub-prime organizations that interacted with clients tended to be even more confused about the GGR [22, 38]. During the Reagan GGR, an abortion provider in Kenya needed clarity on the permissibility of abortion for a woman living with AIDS, and another questioned if a woman verified by a psychologist to be at risk of committing suicide due to an unwanted pregnancy classified as a case of life endangerment [19]. One organization in Brazil was confused about whether partners advocating for liberal abortion laws could be invited to workshops and receptions, and staff in Bangladesh did not know what abortion research was allowed [19].

### Loss of funding

Twenty-one articles discussed either GGR-associated loss of funding or the outcomes of direct or projected funding loss. International Planned Parenthood Federation (IPPF) [31] and Marie Stopes International (MSI) are prime partners who have not complied with any iteration of the GGR, resulting in the recurrent loss of U.S. funding [25]. During the Reagan GGR, IPPF/London’s abortion-related work accounted for approximately US$400,000 annually, though the organization’s rejection of the GGR caused them to lose about US$11 million [26, 38]. During the G.W. Bush GGR, IPPF lost about $18 million in U.S. aid annually and consequently had to cut funding to its affiliates, who are sub-grantees. The sub-grantee Family Planning Association of Kenya (FPAK) lost 58% of its budget, and Planned Parenthood Association of Ghana (PPAG) lost 54% [34], or US$200,000 of funding [39]. Family Planning Association of Nepal (FPAN) lost US$100,000 in direct funding and US$400,000 worth of contraceptive supplies [20], and Family Guidance Association in Ethiopia (FGAE) lost close to half a million U.S. dollars [33, 51]. Organizations that lost funding had to restructure by reducing salaries and laying off staff members [20, 43].

Under the Reagan and G.H.W Bush GGR from 1984 to 1993, the U. S government committed to maintaining its level of family planning aid by reallocating the funds denied to non-compliant organizations to those in compliance with the policy [38]. Documentation of this reallocation remains inaccessible despite a 1991 congressional hearing during which USAID reported that reprogramming notifications would be made publicly available [26, 38]. Under the G.W. Bush GGR, USAID did not provide information on how the policy was implemented [25]. One study reveals that during the G.W. Bush policy years, there was a GGR-associated three to 6 % reduction in U.S. international family planning aid [18]. The most adverse impact on funding was experienced in sub-Saharan African countries [50].

### The chilling effect

The “chilling effect” of the GGR refers to when organizations or health care providers restrict their activities beyond what is required by the policy in order to protect themselves from being accused of non-compliance. In various documented cases, in order to be cautious, providers failed to deliver health services permissible under the policy [23, 41]. In Bangladesh and Turkey, some providers also stopped sharing information on menstrual regulation, and frustrated long-term clients stopped seeking other family planning services that could have benefitted them [19].

Health providers in Egypt ceased all discussions about sepsis after an unsafe abortion, even when this was a major public health concern [19]. An organization in Zambia removed emergency contraception content from its contraception brochure [25]. Some compliant organizations intentionally avoided working with, or requesting proposals from, partners who were not, or likely would reject, complying with the GGR [19, 21]. Others feared even being associated with abortion services, such as a USAID-funded family planning organization in Asia that refused to sell sterilization equipment to a legal abortion clinic, despite the fact that this would not have violated policy requirements [21].

### Impact on advocacy and coalition spaces

In many countries, the GGR hindered efforts to liberalize and implement abortion laws. During the G.W. Bush administration, the same organizations effectively implementing U.S.-funded reproductive health projects in Nepal [7, 43] and Peru [41] had been at the forefront of liberalization advocacy. Organizations in Ethiopia, Kenya, Mozambique, Nigeria, and Uganda had initiatives attempting to reform restrictive abortion laws, and received significant U.S. family planning assistance [40]. As a condition of keeping their funding for crucial programs and service provision, the aforementioned organizations were excluded from abortion reform conversations. The GGR also muted the voices of advocates for liberal abortion laws in Kenya and Ethiopia, while anti-choice groups had no such silencing [29, 42].

In Peru, the GGR amplified anti-choice groups’ narrative against emergency contraception, which resulted in USAID/Peru excusing itself from providing emergency contraception in the country [23]. In Uganda, on the directive of the Catholic cardinal, the government banned emergency contraception across the nation [42].

The GGR also undermined collective advocacy and clinical work during both the Reagan [19] and G.W. Bush [23, 25, 42, 45] policy years as coalitions were often made up of both GGR-compliant and non-compliant organizations. During the Reagan GGR, organizations in Bangladesh that supported menstrual regulation had to fracture their relationships with organizations that did not, which effectively hindered collaborative efforts to promote family planning [19]. Fifteen organizations in Bolivia had banded together to lobby the government on the high national unsafe abortion rate and under G.W. Bush, four of them had to resign due to GGR-related budget threats [33]. The U.S. was the primary donor for the Reproductive Health Response Conflict (RHRC) Consortium, a network of organizations including MSI, which addressed reproductive health for refugees and displaced populations. In 2003, after the GGR was extended to funding from the Department of State, the U.S. ceased RHRC financing [45].

The GGR presented the false choice of continuing to receive funding for programs and services or continuing advocacy work, skewed the debate on abortion and emergency contraception, and fractured partnerships and their collective power to influence change [45].

### Impact on HIV and AIDS

The GGR dismantled efforts to provide comprehensive HIV and AIDS prevention, testing, and treatment. In the early years of the G.W. Bush policy era, confusion about policy restrictions led various organizations to cease their HIV and AIDS work in Ethiopia, including the provision of services that were not subject to the GGR [51]. Later during this policy era, the President’s Emergency Plan for AIDS Relief (PEPFAR) was conceived and exempt from the GGR. Despite this modification, the current expanded GGR does impact PEPFAR funding.

The GGR undermined HIV service provision by organizations that had integrated family planning and HIV and AIDS efforts [25, 26, 29, 44]. Under G.W. Bush, the GGR affected family planning services like condom education, supply, and distribution, all of which were crucial for HIV prevention [51–53]. After GGR-related funding loss, FPAK and MSI-Kenya curtailed their voluntary counseling and testing (VCT) and HIV prevention services [20].

Due to the GGR, organizations in Uganda were forced to separate abortion from HIV and AIDS services, creating vulnerability for women living with HIV who had unwanted pregnancies [29]. The GGR forced organizations supplying comprehensive, integrated services to choose between silos of either family planning or HIV and AIDS service provision [29].

### Impact on abortion

Three studies have quantified the association between the G.W. Bush-era GGR and induced abortion rates [4, 5, 48]. Bendavid et al. (2011) examined the association between 20 sub-Saharan African countries’ exposure to the GGR and induced abortion in women of reproductive age, between 1994 and 2008. Countries that received U.S. financial assistance above a calculated median level were considered to have high GGR exposure. Women in these countries had two and a half times the likelihood of having an induced abortion, compared to women in low-GGR-exposed countries [5].

In a second publication, Jones (2011) evaluated the impact of the policy on induced abortion rates and child health outcomes in Ghana by comparing two periods during which the GGR was in effect (under Reagan and G.W. Bush) to two in which it was not [4]. When the GGR was in effect, abortion rates did not decrease for any demographic, and women living in rural areas had one and a half times the odds of having an induced abortion, compared to women living in urban areas.

A third study implemented the methodology from Bendavid et al. on a global analysis of the association between exposure to the GGR and induced abortion rates [48]. Women in high-exposed Latin American and Caribbean countries had three times the odds of having an induced abortion, compared to women in low-exposed countries. In sub-Saharan Africa, the projections were similar to those found in the Bendavid et al study, with women in high-exposed countries having two times the odds of undergoing an induced abortion, compared to women in low-exposed countries [48]. Together, the available quantitative evidence reveals that GGR implementation was associated with increases in abortion rates, which may be attributable to GGR-based reductions in family planning aid [5] and subsequent reductions in family planning services.

### Impact on contraception and family planning

GGR-related funding losses led to reductions in, or entire shutdowns of, family planning activities and outreach programs. Under the G.W. Bush administration, USAID reduced or stopped contraceptive supplies to 16 countries in sub-Saharan Africa, Asia, and the Middle East [43]. The Lesotho Planned Parenthood Association (LPPA), the only distributor of condoms in the country, did not receive U.S. condom supplies for almost eight years [43]. “Condom corners” that supplied free condoms to rural communities in Ethiopia, Ghana, and Kenya closed down, resulting in contraceptive supply shortages [6, 39, 40]. MSI decreased services and closed clinics in Kenya [6, 20, 33], Tanzania [52], Uganda [33], and Zimbabwe [53]. IPPF closed down clinics in the Democratic Republic of the Congo, Ethiopia, Ghana, Kenya, Zambia, and Zimbabwe [6, 8, 20, 39, 51, 53]. Planned Parenthood Association of Ghana closed 57% of their clinics, and rural areas in Ghana experienced a 45% drop in community-based distribution of contraceptive supplies [34]. Some health facilities offering a range of integrated services, including family planning, were the only providers of primary health care, so their closure dissolved communities’ only contact with the health system [50].

From 2001 through 2008, the family planning funding that IPPF lost could have prevented 36 million unintended pregnancies and 15 million induced abortions [43]. Dismantling family planning programs triggers the decrease in contraceptive supplies [34] and modern contraceptive use [5], and an associated increase in unintended pregnancies [4]. Jones’ study revealed an association between GGR-related funding loss and an estimated 12 % increase in rural pregnancies and 500,000 to 750,000 additional unintended births, which may be attributable to the reduction of the community-based distribution of contraceptive supplies [4].

### Impact on maternal and child health

Jones’ estimations reveal that children born from unintended pregnancies related to GGR exposure had poorer health status on height- and weight-for-age indicators when compared to their siblings [4]. Additionally, a master’s thesis found that under G.W. Bush, GGR exposure in Ghana had negative effects on prenatal care access for both rural and urban populations [24], which could have been linked to the shutdown of facilities run by organizations like MSI [39]. Bingenheimer & Skuster (2017) hypothesize that the negative outcomes of the GGR implementation, including an increase in unsafe abortions and decrease in health system access, could likewise have negative repercussions on maternal morbidity and mortality [11].

## Discussion

To our knowledge, this is the first comprehensive scoping review to track and coalesce the impacts of the GGR from its inception to 2017. This review provides a preliminary mapping of the vast impacts of the policy across health systems, which researchers and policymakers can use as the first step in their GGR work. This review also reveals that the GGR is a poorly constructed and implemented policy (Table 5).

**Table 5:** Prime Partners and Sub-grantees

A “prime partner” is an organization that receives U.S. funding directly from the U.S. government. Both U.S.-based NGOs and foreign NGOs can be prime partners. All U.S. funding and policy requirements are passed down from prime partners to their sub-grantees.	
A “sub-grantee,” “sub-recipient,” or “sub-prime” is an organization that receives U.S. funding from a prime partner, rather than directly from the U.S. government. Sub-grantees are one step removed from a direct relationship with the U.S. government, and communications about their funding are filtered through the prime partner.	

Public policy literature demonstrates the crucial importance of preparation and planning when creating [54] and implementing policies [55, 56]. Decision-making on the content of the GGR neglected to consider all the actors who would be involved with the policy’s implementation, as evidenced by the resulting miscommunication and misunderstanding on compliance requirements. Studies have shown that when critical stakeholders are excluded from agenda-setting and/or the policy formulation process [57], desired policy outcomes may fail to emerge [58, 59]. In the scoped literature, there is no evidence to suggest that organizations to whom the policy applies were present when crafting the Standard Provisions, and a plethora of evidence reveals that the policy does not have its stated intended outcome of reducing rates of abortion and saving lives.

GGR decision-makers have not given adequate attention to the contextual understanding necessary for implementing the health system changes mandated by the policy [60], which may partially explain the miscommunication between U.S. prime partners and their sub-grantees (Table 6). Prime partners operating at the macro-level of the health system may understand what policy compliance entails because they have direct communication with the U.S. government. Sub-grantees at the meso level of the health system are implementing GGR-constrained services without having direct contact with the U.S. government and may be less informed about the GGR. The health care providers operating at the micro-level of the health system have to make decisions informed by the GGR, and yet they are so far removed from policy compliance standards. When the multiple and interacting levels of the health system must confront the GGR, there is ample opportunity for miscommunication, confusion, and chilling effects. For example, in a country like South Africa, in which abortion is permitted upon request [61], imposing the GGR generates confusion and fear as providers negotiate between local law and GGR compliance.

**Table 6:** The GGR- a poorly constructed and implemented policy

• There is no available documentation of all the actors involved in crafting the GGR.	
• Preparation and planning for the implementation of the GGR are generally poor.	
• Confusion about the GGR presents differently for stakeholders at the micro, meso, and macro levels of the health system.	
• Implementation of the GGR takes a top-down approach with no bottom-up input.	
• Since its inception, the GGR has had harmful impacts on more than just family planning, including miscommunication and misunderstanding of policy mandates; segregation of integrated systems; loss of funding and staff; gaged advocacy; disrupted health delivery systems; and reduced health service provision.	

The recent expanded GGR worsens the confusion surrounding this policy as it also applies to non-family planning global health stakeholders. In 2003, President G.W. Bush authorized PEPFAR to spend up to US$15 billion over five years to address HIV and AIDS, tuberculosis (TB), and malaria [62]. In its first four years, PEPFAR reduced AIDS-related deaths by about 10.5% [63] and has supported the provision of antiretroviral therapy (ART) for about 14.6 million people since its inception [64, 65]. When G.W. Bush issued a presidential memorandum to reinstate the GGR, it specified that the policy would not apply to PEPFAR funding. In 2017, President Trump issued a presidential memorandum to reinstate and expand the GGR, which no longer excludes funding through PEPFAR, threatening almost one and a half decades of progress combating HIV and AIDS. Newly published research indicates that the GGR is already harming PEPFAR efforts [16, 66]. Potential financial impacts of the GGR on programs like PEPFAR that include education and prevention of HIV and AIDS may mean that more resources will be needed for treatment.

Although the quantitative studies investigating the association between the GGR and abortion rates debunk the claim that the GGR reduces abortion incidence [4, 51], empirical evidence has been disregarded in the policy-making. The evidence on the GGR has consistently revealed how the policy is rupturing effective integrated services [28] and in some instances, leaving entire communities without clinic access [36, 41]. This scoping review has provided evidence that the GGR is dismantling health systems by causing confusion about its practical implementation; unraveling integrated systems; diminishing qualified staff and crucial resources; silencing necessary advocacy voices and spaces; and reducing health service provision – including but not limited to family planning services – as well as health outcomes indicators. Policymakers can use the findings in this review to create policies based on evidence in order to effectively achieve their intended outcomes.

### Avenues for future research

Knowledge of the conditions underpinning policy compliance or non-compliance is a small fraction of comprehending the GGR. More research and policy analysis are needed to understand the organizational processes and the health systems to which the GGR gets applied to ultimately explain why desired policy outcomes failed to emerge or why the unintended and harmful impacts of the GGR occurred. This evidence would be invaluable for GGR policy reform.

In order to mitigate policy harm, more empirical research is needed to understand the confusion surrounding the GGR at the individual, community, and national or global levels of the health system. More research is also needed to track and explore changes in domestic policies as a response to or consequence of the GGR.

### Limitations

The search strategy included only articles published in English. This strategy poses a potential limitation if relevant works in other languages were removed. The majority of the literature in this review is grey and has limited discussion and presentation of the methodology. Given the methodological constraints, the results of this scoping review should be cautiously interpreted. For example, few of the studies [4, 8, 51] used population data to explore the association between the GGR and abortion rates. There is a scarcity of abortion data, especially in countries in which it is criminalized and reporting systems may not exist [67].

## Conclusion

The evidence shows that even before recent expansion and reinstatement of the GGR, the previous iterations of the policy deteriorated health system functions beyond family planning programs. At the micro-level, provider-client interactions were affected as health care providers could not share the full range of reproductive information and options. At the meso-level, civil society was silenced from abortion advocacy. At the macro-level, coalition spaces dissolved and entire organizations lost funding, which had crippling effects for beneficiaries of health services, organizational functions, and health systems as a whole.

The policy’s development and implementation processes are flawed, and the consequences of these flaws are experienced by low- and middle-income countries (LMICs) who are beneficiaries of U.S. foreign assistance. Policy analysis and more empirical research that investigates the interactions of the policy’s impact at all levels of the health system would generate the evidence needed to change the conditions of the GGR and mitigate its harms.

## Data Availability

The search strategies generated for this review are available from the corresponding author upon reasonable request. An example of the strategy for Pubmed searches is also available.

## Appendix

**Table 7:** Search Terms & Key Words per Focus Area

Key Topics	MeSH	key search words (keywords will be searched TIAB search filter)	Search strategy per topic (PubMed)
Global Gag Rule	none	“Global Gag Rule” OR “GGR” OR “Mexico City Policy” OR “Protecting Life in Global Health Assistance”	((Global Gag Rule) OR Mexico City Policy) OR Protecting Life in Global Health Assistance
Abortion	“Abortion, Induced” OR “Abortion, Septic” OR “Abortion, Criminal”	abortion	(((abortion) OR “Abortion, Criminal”[Mesh]) OR “Abortion, Septic”[Mesh]) OR “Abortion, Induced”[Mesh]
Advocacy	“consumer advocacy”	Advocacy	Advocacy
Commodities (male & female condoms; PrEP; Emergency Contraception: PEP)	“Condoms” OR “Condoms, Female” OR “Pre-Exposure Prophylaxis” OR “Contraceptives, Postcoital” OR “Post-Exposure Prophylaxis”	condoms OR female condoms OR male condoms OR Emergency contraceptives OR EC OR Pre-exposure Prophylaxis OR PrEP OR post-exposure prophylaxis OR PEP	(((((“Condoms”[Mesh]) OR “Condoms, Female”[Mesh]) OR “Pre-Exposure Prophylaxis”[Mesh]) OR “Contraceptives, Postcoital”[Mesh]) OR “Post-Exposure Prophylaxis”[Mesh]) OR ((((((((condoms) OR female condoms) OR male condoms) OR pre-exposure prophylaxis) OR PrEP) OR emergency contraceptives) OR EC) OR post-exposure prophylaxis)
Family Planning	“Family Planning Services” OR “Family Planning Policy” OR “Pregnancy, Unplanned” OR “Pregnancy, Unwanted” OR “Sex Education”	“unintended pregnancy” OR “unwanted pregnancy” OR family planning	((((“Family Planning Services”[Mesh]) OR “Family Planning Policy”[Mesh]) OR “Pregnancy, Unplanned”[Mesh]) OR “Pregnancy, Unwanted”[Mesh]) OR (((((unintended pregnancy) OR unwanted pregnancy) OR unplanned pregnancy) OR family planning))
Gender-based Violence	Domestic Violence OR Intimate Partner Violence OR Sex Offenses	gender-based violence OR domestic violence OR Intimate Partner Violence OR Sex Offenses	gender-based violence
Global Health Assistance	“United States Government Agencies” OR “Federal Government” OR “United States Agency for International Development” OR “Center for Disease Control and Prevention” OR “US Department of Defense” OR “Peace Corps”	“US foreign assistance” OR “Global Health Assistance”	((“United States Government Agencies”[Mesh]) OR “Federal Government”[Mesh]) OR “United States Agency for International Development”[Mesh]
HIV/AIDS & STIs	“HIV” OR “Acquired Immunodeficiency Syndrome” OR “Sexually Transmitted Diseases”	sexually transmitted infections OR Sexually transmitted Diseases OR HIV/AIDS OR HIV OR AIDS OR STD OR STI	(((“HIV”[Mesh]) OR “Acquired Immunodeficiency Syndrome”[Mesh]) OR “Sexually Transmitted Diseases”[Mesh]) OR sexually transmitted infections
Human Rights	“Human Rights” OR “Human Rights Abuses”	human rights	(((“Human Rights”[Mesh]) OR “Human Rights Abuses”[Mesh])) OR Human rights
Infectious Diseases	“Communicable Diseases” OR “Tuberculosis” OR “Malaria” OR “Zika Virus” OR “Zika Virus Infection” OR “Hemorrhagic fever, Ebola”	Infectious Diseases OR Tuberculosis OR Malaria OR Zika OR Ebola	((((((Infectious Diseases) OR (((tuberculosis) OR malaria) OR zika)) OR “Zika Virus Infection”[Mesh]) OR “Zika Virus”[Mesh]) OR “Malaria”[Mesh]) OR “Tuberculosis”[Mesh]) OR “Communicable Diseases”[Mesh]
Key Populations	“Sexual Minorities” OR “Sex Workers” OR “Adolescent” OR “Substance Abuse, Intravenous” OR “Prisoners”	Lesbian’ OR ‘Gay’ OR ‘Bisexual’ OR ‘Transexual’ OR ‘Transgender’ OR ‘Queer’ OR ‘Intersexual’ OR ‘LGBTQI’ OR ‘Men who have Sex with Men’ OR ‘MSM’ OR ‘Sex Workers’ OR ‘HIV key populations’ OR ‘adolescent girls’ OR’ young women’ OR ‘AGYW’ OR ‘people who inject drugs’ OR PWID OR Prisoners	(((((HIV Key Populations) OR “Sexual Minorities”[Mesh]) OR lesbian) OR “Sex Workers”[Mesh]) OR “Adolescent”[Mesh]) OR (((((((((((((((gay) OR bisexual) OR transexual) OR Queer) OR Intersexual) OR LGBTQI) OR Men who have sex with Men) OR MSM) OR Sex Workers) OR HIV key populations) OR Adolescent Girls) OR Young Women) OR AGYW) OR People who inject drugs) OR PWID)
Maternal and Child Health	“Maternal-Child Health Centers” OR “Maternal-Child Health Services” OR “Maternal Health Services” OR Maternal Health	Maternal and Newborn Child Health’ OR ‘Maternal and Child Health’	(((“Maternal-Child Health Centers”[Mesh]) OR “Maternal-Child Health Services”[Mesh]) OR (maternal and newborn child health)) OR ((maternal and child health))
Maternal Mortality & Morbidity	“Maternal Mortality” OR “Maternal Death”	Maternal mortality’ OR maternal morbidity	((“Maternal Mortality”[Mesh]) OR maternal mortality) OR maternal morbidity
Non-Communicable Diseases	“Cholera” OR “Uterine Cervical Neoplasms”	Cholera’ OR ‘Cervical Cancer’ OR non-communicable diseases OR ‘HPV’ [TIAB]	((“Cholera”[Mesh]) OR “Uterine Cervical Neoplasms”[Mesh]) OR (((cervical cancer) OR cholera) OR non-communicable diseases)
Orphans & Vulnerable Children	“Child, Orphaned” OR “Child, Abandoned”	Orphans and vulnerable children [TIAB]	orphans and vulnerable children
Prevention of Maternal To Child Transmission	none	PMTCT [TIAB] OR ‘Prevention of Mother to Child Transmission’	(Prevention of Mother to Child Transmission) OR PMTCT
Reproductive Health	“Reproductive Health” OR “Reproductive Health Services” OR “Reproductive Rights”	TIAB- reproductive health OR reproductive health services OR reproductive rights OR sexual and reproductive health and rights OR SRHR	(((“Reproductive Health”[Mesh]) OR “Reproductive Health Services”[Mesh]) OR “Reproductive Rights”[Mesh]) OR “Sex Education”[Mesh]
Water, Sanitation, and Hygiene	“Hygiene” OR “Sanitation”	‘water, sanitation and hygiene’ [TIAB] OR WSH [TIAB] OR sanitation [TIAB] or Hygiene [TIAB]	water sanitation and hygiene

## Abbreviations

AIDS: Acquired Immunodeficiency Syndrome
ART: Antiretroviral Therapy
CHANGE: Center for Health and Gender Equity GBV Gender-based Violence
GGR: Global Gag Rule
HIV: Human Immunodeficiency Virus
IPPF: International Planned Parenthood Federation
LMICs: Low- and Middle-Income Countries
MCH: Maternal and Child Health
MCP: Mexico City Policy
MSI: Marie Stopes International
NGO: Non-governmental Organization
PEPFAR: President’s Emergency Plan for AIDS Relief
PLGHA: Protecting Life in Global Health Assistance
SRHR: Sexual and Reproductive Health and Rights
TB: Tuberculosis
USAID: United States Agency for International Development
VCT: Voluntary Counseling and Testing
WASH: Water, Sanitation and Hygiene

## Footnotes

1 The “passive referral” exception permits a health care provider to inform a woman where she can obtain a legal abortion, if all of the following criteria are met: A pregnant woman clearly states that she has already decided to have a legal abortion and asks where one can be obtained, and the provider believes that a response is required based on the ethics of the country’s medical profession.

2 The Center for Health and Gender Equity (CHANGE) is a U.S.-based non-governmental organization whose mission is to promote sexual and reproductive health and rights (SRHR) as a means to achieve gender equality and empowerment of all women and girls by shaping public discourse, elevating women’s voices, and influencing the United States Government.
